# A quantum annealing approach to ionic diffusion in solids

**DOI:** 10.1038/s41598-021-86274-3

**Published:** 2021-03-31

**Authors:** Keishu Utimula, Tom Ichibha, Genki I. Prayogo, Kenta Hongo, Kousuke Nakano, Ryo Maezono

**Affiliations:** 1 School of Materials Science, JAIST, Asahidai 1-1, Nomi, Ishikawa 923-1292 Japan; 2 School of Information Science, JAIST, Asahidai 1-1, Nomi, Ishikawa 923-1292 Japan; 3 Research Center for Advanced Computing Infrastructure, JAIST, Asahidai 1-1, Nomi, Ishikawa 923-1292 Japan; 4 International School for Advanced Studies (SISSA), Via Bonomea 265, 34136 Trieste Italy

**Keywords:** Mathematics and computing, Physics

## Abstract

We have developed a framework for using quantum annealing computation to evaluate a key quantity in ionic diffusion in solids, the correlation factor. Existing methods can only calculate the correlation factor analytically in the case of physically unrealistic models, making it difficult to relate microstructural information about diffusion path networks obtainable by current ab initio techniques to macroscopic quantities such as diffusion coefficients. We have mapped the problem into a quantum spin system described by the Ising Hamiltonian. By applying our framework in combination with ab initio technique, it is possible to understand how diffusion coefficients are controlled by temperatures, pressures, atomic substitutions, and other factors. We have calculated the correlation factor in a simple case with a known exact result by a variety of computational methods, including simulated quantum annealing on the spin models, the classical random walk, the matrix description, and quantum annealing on D-Wave with hybrid solver . This comparison shows that all the evaluations give consistent results with each other, but that many of the conventional approaches require infeasible computational costs. Quantum annealing is also currently infeasible because of the cost and scarcity of qubits, but we argue that when technological advances alter this situation, quantum annealing will easily outperform all existing methods.

## Introduction

The quantum annealing technique^1,2^ has been widely and successfully applied to challenging combinatorial optimizations^3^, including NP(Non-deterministic Polynomial time)-hard^4^ and NP-complete problems^3,5–7^. Realistic problems such as the capacitated vehicle routing problem (CVRP), optimization of traffic quantity^8–11^, investment portfolio design^12^, scheduling problems^13^, and digital marketing^14^ have recently been addressed by quantum annealing. The technique has also been applied to improve the performance of machine learning^15,16^.

In the chemistry and materials science domain, however, relatively few applications have been found, other than investigation of the molecular similarity problem^17^ or the search for protein conformations^18^. This contrasts with the many applications of quantum gate computing to this field^19^, e.g., in quantum phase estimation. This imbalance is self-perpetuating: chemists and materials scientists are unfamiliar with quantum annealing, and so do not think to use it. Finding additional applications of the technique is therefore important not only for the sake of the applications themselves, but also for the sake of increasing recognition of quantum annealing as a useful method in this domain.

In the quantum annealing framework, an optimization problem is mapped into a quantum spin system described by the Ising Hamiltonian^1,2^. The problem is then solved by searching for optimal spin configurations minimizing the energy of the Hamiltonian. In this framework, the problem of finding an optimum in the presence of many local minima is solved by using quantum tunneling (i.e. virtual hopping) to cross high energy barriers. The quantum framework is an increasingly popular tool for the solution of optimization problems in the everyday, classical world. However, its application to problems in the quantum world^17^ seems to be surprisingly rare. In the present study, we applied it to ionic diffusion in solids^20^. This quantum-mechanical topic, which is of great interest in both pure and applied materials science, originally attracted attention in connection with the microscopic analysis of mechanical strengths^21^, and more recently has been connected to the efficiency of batteries, systems where charge-carrying ions diffusing in the solid electrolyte are clearly of central importance^22–24^.

Among the various mechanisms^20^ of ionic diffusion, we concentrate here on the vacancy mechanism^20^, in which ions hop only between lattice sites. Although many ab initio works have provided insight into *microscopically* ’easier paths’ for the ion to hop along, it remains difficult to get practically useful knowledge of the diffusion coefficient *D* as a *macroscopic* quantity. To connect the microscopic knowledge with the macroscopic quantity, we must cope with the difficult problem of counting all possible processes by which an ion is pulled back toward a vacancy^25^ (while also being pulled in other directions, as explained in the next section). This process is described by the *correlation factor*^20,25^ *f*. The evaluation of *f*, which involves identifying the optimum routing as a vacancy hops around on lattice sites for a given anisotropic *easiness*, is essential for connecting the microscopic analysis with the evaluation of practically useful macroscopic quantities^25^. Such a routing problem is analogous to classical ones that have been successfully treated in the annealing framework. Otherwise, the evaluation is far too difficult to solve in the general case; so far, only very limited cases and simple models (e.g., the simple cubic lattice) have been solved^20^. In the present work, we provide a way to formulate the evaluation in the annealing framework, and show that the method successfully overcomes difficulties unsolved by conventional approaches.

## Formulation

### Correlation factor in diffusion mechanism

We consider a form of atomic diffusion where the atom to be considered (the ‘tracer’) hops onto a neighboring vacancy site (‘hole’) generated by thermal processes. Let the tracer be located on a site $\alpha$. At the initial step ($i=0$), we will write $\alpha = S$ (Start). Any hopping of the tracer onto neighboring vacant sites generates a hole on $\alpha = S$ at the $i=1$ step. This hole then becomes a possible vacant site by which the tracer may get back to $\alpha = S$, a process described as ‘the hole pulls the tracer back with a certain probability’. This probability is typically manifest as a reduction of the effective stride of the tracer by a factor *f*, the correlation factor of the diffusion.

**Figure 1:**
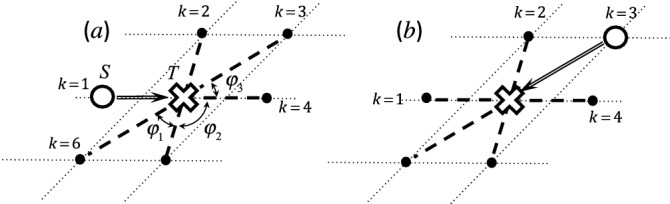
Examples of snapshots for the vacancy (white circles, initially at site *S*) to attract a tracer (white crosses at site *T*) to the vacancy’s position. The horizontal direction to the right is defined to be identical to that of the diffusion flow to be considered. The vacancy is located at one of the *Z* neighboring sites to site *T* (*Z*=6 as an example in the panels) right before exchanging positions with the tracer. The vacancy site is denoted by *k*. The attraction angles from site *k* are $\theta _1 = \pi$, $\theta _2 = \pi - \varphi _2$, $\theta _3 = \varphi _3$, $\theta _4 = 0$, $\ldots$. The panel (**a**) indicates the most likely case that the vacancy pulls behind the tracer and the panel (**b**) indicates that the vacancy pulls forward the tracer after detour movements.

While the simplest picture would be an immediate ’pull-back’ made by a vacancy at $\alpha = S$ when $i=2$, we must take into account further ways a wandering vacancy can attract a tracer when $i\ge 3$. We shall therefore consider the final state (where the vacancy is about to attract a tracer). Let the site $\alpha = T$ be where the tracer is located at step $i=(N-1)$, immediately before it is finally attracted back to the neighboring vacancy. Because this is an exchange process, the vacancy will be located at $\alpha = T$ when $i=N$. To specify the geometry, let $\theta =0$ be the direction of the diffusion flow with a radius vector centered at $\alpha = T$  (Fig. 1). Let the number of neighboring sites to $\alpha = T$ be *Z*, with locations specified by $\theta _k$. A pulling back by a vacancy at $\theta _k$ is then contributing to the diffusion by its projection, $\cos \theta _k$. Letting $P_k$ be the probability distribution to get a vacancy at a specific $\theta _k$ amongst *Z* when $i=(N-1)$, the ’average cosine’ ,

1 $$\begin{aligned} \left\langle {\cos \theta } \right\rangle = \sum \limits _{k=1}^Z {{P_k}\cos {\theta _k}}, \end{aligned}$$

matters to the correlation factor. Further consideration is required to take into account the fact that a pulling-back process itself is also subject to pulling-back. Such multiple processes are finally convoluted into a form^25,26^ as,

2 $$\begin{aligned} f= & {} 1 + 2\sum \limits _{n = 1}^\infty {\left\langle {\cos \theta } \right\rangle ^n} = \frac{ {1+\left\langle {\cos \theta } \right\rangle } }{1-{\left\langle {\cos \theta } \right\rangle }} \ . \end{aligned}$$

With $\theta$ as in Fig. 1, this factor ranges from $f = 0$ ($\theta = \pi$) through $f = 1$ ($\theta = \pi /2$) to $f\rightarrow \infty$ ($\theta \rightarrow 0$).

### Formulation using quantum annealing Hamiltonian

The evaluation of the correlation factor is therefore reduced to the calculation of the averaged projection given in Eq. (1). The mission of the simulations is to provide the probability $P_k$, which is obtained from the trajectories of a vacancy hopping along optimal paths in the given system, i.e., those satisfying the initial [$\alpha = S$ ($i=1$)] and the final [$\alpha = T$ ($i=N$)] conditions: the probability distribution for these trajectories gives $P_k$ at $i=(N-1)$.

The problem of getting the optimum trajectories is well formulated as a routing problem solved by the quantum annealing, as described in the ‘Introduction’ section. To facilitate this approach, we shall introduce Ising spins to describe the time evolution of the position of the vacancy as follows: Let $q_{\alpha ,i}$ take the value 1 when a vacancy is located at the site $\alpha$ at the step *i*, and otherwise take the value 0. The initial (final) condition is then described as $q_{S,1}=1$ ($q_{T,N}=1$). Under these conditions, the annealing framework is capable of providing optimum trajectories when $i=2\sim (N-1)$. The probability that $q_{k,N-1}=1$ corresponds to $P_k$ in Eq. (1). A trajectory is expressed by a spin alignment $\left\{ q_{\alpha ,i}\right\}$ dominated by an Ising Hamiltonian^8–11^:

3 $$\begin{aligned} {\hat{H}}_N= & {} \sum \limits _{\alpha ,\beta } {\sum \limits _{i = 1}^{N - 1} {\left( {{t_{\alpha \rightarrow \beta }}\cdot {q_{a,i}} {q_{\beta ,i + 1}}} \right) } } + {\lambda _2}\sum \limits _{i = 1}^N {{{\left( {\sum \limits _\alpha {{q_{\alpha ,i}} - 1} } \right) }^2}} \nonumber \\&+ {\lambda _3}{\left( {{q_{S,1}} - 1} \right) ^2} + {\lambda _4}{\left( {\sum \limits _{i = 2}^{N - 1} {{q_{\mathrm{{T}},i}}} } -0\right) ^2} + {\lambda _5}{\left( {{q_{T,N}} - 1} \right) ^2}.\end{aligned}$$

The first term describes the hopping of a vacancy between sites, $\alpha \rightarrow \beta$. The hopping amplitude $t_{\alpha \rightarrow \beta }$ corresponds to the probability of the hopping *p*, which scales with a temperature (*T*) dependence $p_{\alpha \rightarrow \beta }\sim \exp {\left( - \Delta E_{\alpha \rightarrow \beta }/T \right) } \sim \exp {\left( - t_{\alpha \rightarrow \beta }/T \right) }$. Here $\Delta E_{\alpha \rightarrow \beta }$ is the barrier energy for the hopping, which can be evaluated by *ab initio* calculations^25^. The amplitude *t* is therefore related to *p* by $t\propto \ln {p}$. The terms with $\lambda _3$ and $\lambda _5$ denote the initial and final conditions as the constraints. The term with $\lambda _2$ expresses the condition that only one vacancy exists over all the sites, i.e., the assumption that we consider a single vacancy contributing to the pulling-back as the primary contribution to *f*, ignoring multiple-vacancy processes as secondary.

This assumption is reasonable except for some cases. Noted that most of the exceptions are in face-centered metallic crystals, where the bi-vacancy process significantly contributes to the self-diffusion when the temperature is higher than 2/3 of the melting temperature^20^. The term with $\lambda _4$ means that the vacancy never exchanges its position with the tracer until $i=N$, as the problem assumes.

### Evaluation of the correlation factor

As a concrete example, consider a $5 \times 5$ lattice in two dimensions:

4 $$\begin{aligned} \left( \begin{array}{l} {{(0,0) \quad (0,1) \quad (0,2) \quad (0,3) \quad (0,4) }}\\ {{(1,0) \quad (1,1) \quad (1,2) \quad (1,3) \quad (1,4) }}\\ {{(2,0) \quad (2,1) \quad (2,2) \quad (2,3) \quad (2,4) }}\\ {{(3,0) \quad (3,1) \quad (3,2) \quad (3,3) \quad (3,4) }}\\ {{(4,0) \quad (4,1) \quad (4,2) \quad (4,3) \quad (4,4) }} \end{array} \right) , \end{aligned}$$

where the entries in the matrix are the site indices. Suppose that a tracer located initially at (2,1) hops onto (2,2), where initially there was a vacancy. We then consider the process by which the tracer is pulled ‘back’ by the vacancy with an angle $\theta _k$ and probability $P_k$ of evaluating the average given by Eq. (1). The process is complete when the vacancy coalesces with the tracer again ($q_{T,N}=1$). Contributions to the summation are not only from the direct ‘pulling back’ ($\theta _k = \pi , N=2$) from (2,1) [the site where a new vacancy appears due to the tracer’s hopping], but also from other possible sites at which the vacancy arrives after strolling for several time steps, as shown in Table 1.

**Table 1:** Possible trajectories for a vacancy generated at (2,1) due to hopping by a tracer.

Trajectory	$\theta$	Contribution	$H_N$
(2,1)(2,2)	*π*	1*t*	$H_2$
(2,1)(1,1)(2,1)(2,2)	*π*	3*t*	$H_4$
(2,1)(2,0)(2,1)(2,2)	*π*	3*t*	$H_4$
(2,1)(3,1)(2,1)(2,2)	*π*	3*t*	$H_4$
(2,1)(1,1)(1,2)(2,2)	*π* /2	5*t*	$H_6$
(2,1)(3,1)(3,2)(2,2)	3*π* /2	5*t*	$H_6$
(2,1)(1,1)(1,0)(2,0)(2,1)(2,2)	*π*	7*t*	$H_8$
.			
(2,1)(1,1)(1,2)(1,3)(2,3)(2,2)	*π* /2	7*t*	$H_8$
.			

The vacancy coalesces with the tracer again after taking $(N-1)$ steps on the 2-dim. lattice shown in Eq. (4). The coalescence angle $\theta$ is measured from the direction of the initial hop by the tracer. Each trajectory contributes to the summation in Eq. (1) with weight $P_k$ corresponding to the energy $E \sim \sum _{\alpha \beta } {t_{\alpha \rightarrow \beta }}$. For this simplified example, we set $t_{\alpha \rightarrow \beta }=t$ (only between nearest neighboring sites). Each trajectory is identified by the annealing simulation using the Hamiltonian $H_N$.

Let us denote the contributions from trajectories obtained by the simulation with the Hamiltonian $H_N$ as

5 $$\begin{aligned} P_k^{(N)} =\sum _{l\in {\Omega };~\mathrm{trajectories}}{\pi _l} \ , \end{aligned}$$

where *l* indexes each trajectory and $\Omega$ is the space formed by all the contributing trajectories. Each contribution from a trajectory with energy $E_l^{(N)}$ would be expressed as $\pi _l\sim \exp {\left( -E_l^{(N)} /T \right) }$. For example, in the case of $N=4$ in Table 1, $\pi _l\sim \exp {(3t)}\sim p^3$. Noticing that trajectories with different *N* values (numbers of steps to arrive at coalescence with a tracer) are mutually exclusive, the probability $P_k$ can be expressed as a sum of each exclusive contribution with different *N*:

6 $$\begin{aligned} {P_k} = \sum \limits _{N = 2}^{{N_{\max }}} {P_k^{(N)}} \ , \end{aligned}$$

where ${P_k^{(N)}}$ is the probability of finding a vacancy at a neighboring site with ${\theta _k}$ obtained from the simulation with ${\hat{H}}_N$. ${P_k^{(N)}}$ is obtained as the ratio of the number of trajectories with ${\theta _k}$ divided by the total number of trajectories within the simulation using ${\hat{H}}_N$.

In the procedure, quantum annealing computers (QACs) are used only to identify the optimal trajectories while the calculation of Eq. (1) is made by a classical counting over a table like Table 1. To get such a table, the annealing simulations should be repeated even within a fixed ${\hat{H}}_N$. Recalling that an annealing simulation gives an optimal trajectory, enough repetition is required to search all the possible trajectories that are likely to be degenerate even within a ${\hat{H}}_N$. After all the possible trajectories have been tabulated, the calculation of Eq. (1) by the classical counting on the table can be attempted. One might wonder whether it is possible to perform the *on-the-fly* evaluation of Eq. (1) during the search for optimal trajectories. For example, suppose that ‘$\theta =0$’ were obtained 5 times, possibilities. One might be tempted to use the frequency of the appearance of a particular angle for an ‘on-the-fly’ calculation of $P_k$. However, this cannot be justified at least for QAC, as we note later in the first paragraph of the ‘Discussion’ section.

## Results and discussion

### Verification of benchmark

For some selected cases with simple lattices, it is possible to describe the multi-scattering processes contributing to the correlation factor in terms of recursion equations, and thus to find analytical solutions^20^; some examples are shown in Table 2.

**Table 2:** Correlation factors *f*, obtained analytically for simple lattice model systems^20^.

Lattice	*f*
Beehive	1/3
2-Dim. tetragonal	0.467
2-Dim. hexagonal	0.56006
Diamond	1/2
Simple cubic	0.6531
Body-centered cubic	0.7272, (0.72149)
Face-centered cubic	0.7815

**Table 3:** The convergence of the correlation factors evaluated by ‘(a) Quantum Annealing with D-wave (QA)’, ‘(b) Simulated Quantum Annealing (SQA)’, ‘(c) Classical Random Walk (CRW)’, and ‘(d) Matrix Updating method (MU)’, depending on the system size *N*.

$N_{\mathrm{max}}$	QA(a)	SQA(b)	CRW(c)	MU(d)
1	–	–	–	–
2	0.600	0.600	0.600	0.600
3	–	–	–	–
4	0.542	0.542	0.542	0.542
5	–	–	–	–
6	0.520$^*$	0.519	0.519	0.519
7	–	–	–	–
8	–	–	0.507	0.507
9	–	–	–	–
10	–	–	0.499	0.499
11	–	–	–	–
12	–	–	0.495	0.494
13	–	–	–	–
14	–	–	–	0.491
	$\cdots$	$\cdots$	$\cdots$	$\cdots$
32	–	–	–	0.477
	$\cdots$	$\cdots$	$\cdots$	$\cdots$
492	–	–	–	0.468
	$\cdots$	$\cdots$	$\cdots$	$\cdots$
502	–	–	–	0.468
	$\cdots$	$\cdots$	$\cdots$	$\cdots$

($^*$: The difference from the other methods is attributed to that our QA calculation could count over only 94.54% of the trajectories, because of the limited number of samples due to the computational cost).

The values given in Table 2 can be used to test our formulation and its implementation. We are able to reproduce the value *f* = 0.467^27^ for a two-dimensional tetragonal lattice by our procedure, as described below. Note that the analytical solution is obtained only for a quite limited case in which the initial and the final positions of the tracer are within one step of each other, ($T=S+1$)^28^, while our treatment is never limited by such toy-model assumption. The present approach is therefore capable of providing interesting knowledge going beyond what can be learned by existing methods.

Though ‘(a) Quantum annealing computers (QAC)’ are ultimately the preferred technology for counting up trajectories to get $P_k$, the availability of such devices is still limited, not only by financial considerations, but also by the total number of qubits technically achieved. As explained later, current availability enables us to try up to $N_{\mathrm{max}}\sim 5$: far too few to verify the calibration of the two-dimensional tetragonal lattice (*f* = 0.467^27^).

As possible substitutes, we can list (b) simulated quantum annealing (SQA)^29,30^/ path integral monte carlo (PIMC)^31,32^’, ‘(c) classical random walk (CRW)’, and ‘(d) matrix updating (MU)’, in order of their closeness to (a). Unfortunately, for larger $N_{\mathrm{max}}$, the feasibility of (b) and (c) proved limited.

For ‘(b) SQA’, the required computational cost is dominated by the annealing time, i.e., the time to decrease the transverse magnetic field. To achieve the equilibrium Boltzmann distribution, this time increases with system size *N* as $\sim \exp (N)$^32^. This limits the possible number of trajectories obtainable at an affordable cost, leading to larger error bars in Eq. (6), as shown in Table 3.

For ‘(c) CRW’, feasibility is assured up to $N_{\mathrm{max}}$=12 in the present case. In this method, the computational time is dominated by the number of stochastic trials. For a step there are *Z* possible ways of hopping to nearest neighboring sites ($Z=4$ in this benchmark case). The total number of possibilities for an *N*-step trajectory amounts to $Z^N$, which easily becomes very large as *N* increases.

By using ‘(d) MU’, we can successfully verify the calibration by going up to $N_{\mathrm{max}}$=500, as described below (Table 3). We introduce the vacancy hopping operator

$$\begin{aligned} {\hat{T}} = \sum \limits _{i,j} {{t_{ij}}\cdot a_i^\dag a_j}, \end{aligned}$$

Consider a field described by the matrix

$$\begin{aligned} {F_0} = \left( {\begin{array}{*{20}{c}} 0&{}\quad 0&{}\quad 0&{}\quad 0&{}\quad 0\\ 0&{}\quad 0&{}\quad 0&{}\quad 0&{}\quad 0\\ 0&{}\quad 0&{}\quad 1&{}\quad 0&{}\quad 0\\ 0&{}\quad 0&{}\quad 0&{}\quad 0&{}\quad 0\\ 0&{}\quad 0&{}\quad 0&{}\quad 0&{}\quad 0 \end{array}} \right), \end{aligned}$$

where each element $(F_{0})_{i,j}$ corresponds to the location of a hopping site. The value ‘1’ in $F_0$ indicates the (initial) location of a vacancy, whereas ‘0’ means the vacancy is absent. We update the field at step *K* to $F_K$, by

7 $$\begin{aligned} {F_K} = {\hat{T}}\cdot {F_{K - 1}} \ . \end{aligned}$$

In the present case (two-dimensional tetragonal lattice), we assume $t_{ij}$ is isotropic and only connects between the nearest neighboring sites. This drives the field matrix to

$$\begin{aligned} {\left( {{F_K}} \right) _{i,j}} = {\left( {{F_{K - 1}}} \right) _{i - 1,j}} + {\left( {{F_{K - 1}}} \right) _{i + 1,j}} + {\left( {{F_{K - 1}}} \right) _{i,j + 1}} + {\left( {{F_{K - 1}}} \right) _{i,j - 1}} \ . \end{aligned}$$

The constraint that the vacancy not coalesce with the tracer until the given final step *N* can be expressed as ${\left( {{F_K}} \right) _{i',j'}}=0$ for $K < N$ where $\left( i',j'\right)$ is the location of the tracer site. After updating the field matrix until step *N*, each matrix element shows how many trajectories being possible to give a vacancy at that site after *N* steps, from which we can evaluate $P_k$ and thus *f*. As shown in Table 3, *f* falls as *N* increases. It is at 0.468 when $N = 500$, and the rate of decline has become very small. Thus, it appears to be asymptotically approaching the value from the analytical solution, 0.467.

The feasibility of ‘(a) Quantum annealing computers (QAC)’ is determined in large part by the available number of qubits, $N_{\mathrm{Qubit}}^{(\mathrm available)}$, currently 2048^33^. The *required* number of qubits scales in the present case as the product of $N_{\mathrm{max}}$ and the size of the lattice ($M\times M$ in the two-dimensional case; $5\times 5$ in the example). Therefore, the maximum possible $N_{\mathrm{max}}^{(\mathrm possible)}$ may be estimated as 81 (= 2048/25); for a user with a practical budget situation, it is probably closer to five. We note however that the computational limitation of being directly and linearly proportional to $N_{\mathrm{Qubit}}^{(\mathrm available)}$ still renders (a) more promising than other methods like (b) and (c).

For ‘(a) QAC’, we used D-Wave^34^ applied to $(N_{\mathrm{max}}+1) \times (N_{\mathrm{max}}+1)$ lattice size for $N_{\mathrm{max}}=2,4,6\ldots$ in order. Since implemented topologies of qubits interconnections (chimera graph) are not capable in general to describe Ising spin couplings as it is in the Hamiltonian, some of the couplings (spins directly couple with each other in the Hamiltonian, say $J_{12}\sigma _1\sigma _2$) are equivalently realized by the synchronized qubits pairs (i.e., $\sigma _1$–$\sigma _2$ in the Hamiltonian is realized as $\sigma _1$–$\tau _1$...$\tau _2$–$\sigma _2$, where $\tau _{1}$ and $\tau _{2}$ are distant but synchronized)^35^. The technique costs the number of qubits than that of pure required one in the model Hamiltonian. Even using 2000 qubits, we could embed our problem only upto $N_{\mathrm{max}}=2$ on the D-wave using the technique. In such a case, we can use the ‘Hybrid solver’ to resolve the problem^36^. The solver itself works on a classical computer, decomposing the original size problem into a set of smaller chimeric graphs those are possible to be handled by D-wave. The set of results by D-wave is then post-processed by the solver to get the answer of the original problem on the classical computer. By using the solver, we have confirmed that proper trajectories are obtained upto, at least, $N_{\mathrm{max}}=12$. However, to get the correlation factor finally, we have to count over all the trajectories, for which we could achieve upto $N_{\mathrm{max}}=6$ due to the D-wave resource limitation. For $N_{\mathrm{max}}$=2, 4, and 6, we sampled 1, 15, and 240 solutions, covering 100%, 100%, and 94.54% of the trajectories, respectively. All the above limitations are, however, coming purely from the technical/implementational aspect of Quantum Annealing machines. It is straightforward for us to make the limitations ahead assisted by the intensive developments on the implementations such as the increasing $N_{\mathrm{Qubit}}^{\mathrm{(available)}}$, improved topologies of chimera graph etc. (e.g., pegasus graph^37^). We note that the intrinsic computational cost for the trajectory sampling is just several $\mu$sec. as we confirmed.

### Discussions

In the procedure explained above, it is assumed that all the degenerate ground state spin configurations (i.e., the optimal trajectories) can be found after a sufficiently large (but finite) numbers of trials of the annealing simulation. We should note, however, that there seems to be no firm theoretical basis for this assumption. In SQA, by contrast, it is guaranteed that all degenerate states will be realized under the Boltzmann distribution if the transverse magnetic field is decreased by the correct procedure^32^. For QAC, we could not find such a clear foundation, but the literature seems to support our assumption. It has been reported that a D-Wave machine can realize the optimal states dominated by the Boltzmann distribution under an ideal operation^38^. There is also a report that, in the setting of quadratic unconstrained binary optimization, Gaussian noise intentionally added on the coefficients *improves* the reproducibility of simulations^35^. If the unsatisfactory reproducibility was due to the ‘bias in the frequency to get equivalent degenerate solutions’, then the *improvement* seems to correspond to a hopeful procedure to ensure our assumption here. It is interesting to estimate how much error will occur in the correlation factor *f* when some degenerate trajectories are missing from the count. Larger multiplicities in the degeneracies occur in the large *N* region, for which MU ($N_{\mathrm{max}} = 501$) is currently the only means of access. We intentionally dropped off some of the degenerate trajectories randomly (at most 10%). The bias in the estimated *f* was then found to be $\sim 0.4$%.

Given the present value of $N_{\mathrm{Qubit}}^{(\mathrm available)}$, MU is still superior to QAC . It is therefore important to discuss what restricts further scalability of MU, and what will make QAC inherently superior when $N_{\mathrm{Qubit}}^{(\mathrm available)}$ is larger. In the space $\Omega$ of all trajectories [mentioned in in Eq. (5)], the weight, $\exp {\left( - E_l^{(N)} /kT \right) }$, dominates only for those trajectories with the most stable energy $E_0^{(N)}$ at lower temperature. Denoting the space formed by such (possibly degenerate) trajectories with the lowest energy as ${\mathcal {A}}\subset \Omega$, then

$$\begin{aligned} P_k^{(N)}\sim \sum _{l\in {{\mathcal {A}}}}{\pi _l}, \end{aligned}$$

for the temperature range. The advantage of QAC in optimization problems in general is its quite efficient ability to extract ${\mathcal {A}}$ from $\Omega$. MU, on the other hand, is a scheme which surveys all the elements of $\Omega$, since it accumulates the number of visits $N_{\mathrm{visits}}$ by the vacancy to every lattice site. When the system size is very large, $|{\mathcal {A}}| \ll |\Omega |$, and hence QAC will perform more efficiently than MU in evaluating $P_k^{(N)}$. From this viewpoint, the present benchmark, the two-dimensional tetragonal lattice, would be highly inappropriate for showing the superiority of QAC for the following reason: In the simplified case ($t_{\alpha \rightarrow \beta }=t$), all the trajectories having the same *N* have the same energy and are elements of ${\mathcal {A}}$. Hence ${\mathcal {A}}= \Omega$ and the advantage of QAC disappears.

MU can easily be generalized to higher dimensional lattices with general shapes and with anisotropic hopping. The temperature dependence of the hopping can be parameterized via the factor $\exp {\left( - E_l^{(N)} /kT \right) }$, and then the scheme would be useful for analyzing temperature-depending diffusion (as would QAC). In the case of the two-dimensional tetragonal lattice, however, the success of MU with $N_{\mathrm{max}}\sim 500$ is in fact just a lucky accident due to the presence of an especially efficient data structure valid only for this case. The factor dominating $N_{\mathrm{max}}$ in MU comes from the upper limit of the largest possible exponent of $N_{\mathrm{visits}}$, represented by various numeric data types. It increases explosively in factorial manner as *N* increases, and (using integer type) easily overflows. In the present work, we use instead the double precision type with mantissa/exponent representation, and find the upper limit of the exponent corresponds to $N_{\mathrm{max}}\sim 500$ even using the simplest possible data structure to store $N_{\mathrm{visits}}$. When we try more general cases, such as three-dimensional lattices, we cannot use such a simple data structure but instead must use ’struct’ type to store $N_{\mathrm{visits}}$, leading to a much reduced $N_{\mathrm{visits}}\sim 20$ (for the three-dimensional cubic lattice).

The difficulty of accommodating $N_{\mathrm{visits}}$ in a practical size of data storage comes from the fact that MU has to treat all the trajectories in $\Omega$. QAC, on the other hand, has no such inherent problem, because it only deals with ${\mathcal {A}}$. The method is then potentially feasible in the future when $N_{\mathrm{Qubit}}^{\mathrm{available}}$ increases.

It is unavoidable, but due to the limitations of available number of qubits at present, the benchmark verification with smaller *N* is not fully appealing to show the benefits of quantum annealing. The cost function of our formalism is to search for the path achieving the highest cumulative hopping probability, $\prod _{\mathrm{path}} \exp { \left( - \Delta E_{\alpha \rightarrow \beta } /kT \right) }$, but in the above verification benchmark, it reduces to a search for the shortest path, being a case with less attraction because of the special condition where all hopping probabilities are identical. However, the framework demonstrates its true power when the hopping probability gets inhomogeneous and especially for a larger *N*. Under such condition, the optimal path achieving the highest hopping probability could take a longer distance, being difficult to be found without the real power of quantum annealing. Since the problem is not only to find the optimal path for each fixed *N* but to integrate over solutions with different *N*, it is critical to identify each optimal path as fast as possible, which makes the use of quantum annealing inevitable. In practical applications, the temperature dependence of the hopping probability generates huge varieties of path networks, which provides further applications of the quantum annealing technique to interesting problems.

## Conclusion

We developed a framework to evaluate the correlation factor, a key quantity used to derive the macroscopic diffusion coefficient for ions in solid materials. The coefficient describes the process by which a vacancy attracts back a tracer even after repeated scattering events. Direct counting of the possible processes is not feasible with conventional computational tools, so the coefficient has previously only been evaluated in limited model cases where simple assumptions allowing the process to be described in terms of recursion formulae can be justified. This has hampered the utilization of microscopic information obtained by ab initio approaches (vacancy rate, formation energy for a defect, energy barrier to hopping, etc.) in macroscopic calculations. By using our framework, we verified as a calibration that direct counting reliably reproduces the results obtained previously by the recursion model. The framework promises to be especially valuable when implemented on quantum computers with the increased number of available qubits made possible by recent technological advances. The applicability of the direct counting approach is never restricted to special cases, so we can investigate how the diffusion coefficient is affected by nano-level tuning of materials and other factors evaluated by ab initio calculations, factors not previously considered applicable to practical ionic hopping networks in realistic materials.
